# The deubiquitylase USP9X controls ribosomal stalling

**DOI:** 10.1083/jcb.202004211

**Published:** 2021-01-28

**Authors:** Anne Clancy, Claire Heride, Adán Pinto-Fernández, Hannah Elcocks, Andreas Kallinos, Katherine J. Kayser-Bricker, Weiping Wang, Victoria Smith, Simon Davis, Shawn Fessler, Crystal McKinnon, Marie Katz, Tim Hammonds, Neil P. Jones, Jonathan O’Connell, Bruce Follows, Steven Mischke, Justin A. Caravella, Stephanos Ioannidis, Christopher Dinsmore, Sunkyu Kim, Axel Behrens, David Komander, Benedikt M. Kessler, Sylvie Urbé, Michael J. Clague

**Affiliations:** 1 Department of Molecular Physiology and Cell Signaling, Institute of Systems, Molecular and Integrative Biology, University of Liverpool, Liverpool, UK; 2 Target Discovery Institute, Nuffield Department of Medicine, University of Oxford, Oxford, UK; 3 FORMA Therapeutics, Watertown, MA; 4 Cancer Research UK Therapeutic Discovery Laboratories, London Bioscience Innovation Centre, London, UK; 5 Adult Stem Cell Laboratory, Francis Crick Institute, London, UK; 6 Ubiquitin Signalling Division, The Walter and Eliza Hall Institute of Medical Research, Parkville, Victoria, Australia; 7 Department of Medical Biology, University of Melbourne, Melbourne, Victoria, Australia

## Abstract

When a ribosome stalls during translation, it runs the risk of collision with a trailing ribosome. Such an encounter leads to the formation of a stable di-ribosome complex, which needs to be resolved by a dedicated machinery. The initial stalling and the subsequent resolution of di-ribosomal complexes requires activity of Makorin and ZNF598 ubiquitin E3 ligases, respectively, through ubiquitylation of the eS10 and uS10 subunits of the ribosome. We have developed a specific small-molecule inhibitor of the deubiquitylase USP9X. Proteomics analysis, following inhibitor treatment of HCT116 cells, confirms previous reports linking USP9X with centrosome-associated protein stability but also reveals a loss of Makorin 2 and ZNF598. We show that USP9X interacts with both these ubiquitin E3 ligases, regulating their abundance through the control of protein stability. In the absence of USP9X or following chemical inhibition of its catalytic activity, levels of Makorins and ZNF598 are diminished, and the ribosomal quality control pathway is impaired.

## Teaser

Clancy et al. develop a specific chemical inhibitor of USP9X and characterize its effects upon the cellular proteome. This analysis reveals a central role in the regulation of ribosomal stalling through control of critical E3 ligases.

## Introduction

Prompt sensing and resolution of aberrant protein translation is essential for the maintenance of protein homeostasis. Several circumstances can give rise to stalled ribosomes, such as insufficiency of a cognate acylated tRNA, defective mRNA, or faulty ribosomes (Arthur and Djuranovic, 2018; Ishimura et al., 2014). The most common cause of ribosomal stalling is thought to be the translation of poly(A), when a nascent mRNA is inappropriately polyadenylated within its coding region to generate a “non-stop” mRNA transcript lacking a stop codon (Arthur et al., 2015; Ozsolak et al., 2010). If a ribosome stalls during translation, it risks being rear-ended by a trailing ribosome. This collision generates a stable di-ribosome complex with a defined structure, which is resolved by the engagement of a dedicated machinery (Juszkiewicz et al., 2018; Collart and Weiss, 2020). In such cases, the E3-ligase ZNF598 ubiquitylates 40S complexes at specific sites on eS10 and uS10 subunits at the di-ribosome interface (Garzia et al., 2017; Juszkiewicz et al., 2018; Juszkiewicz and Hegde, 2017; Sundaramoorthy et al., 2017). This prevents further translation and initiates quality control processes (e.g., degradation of the associated mRNA) and ribosomal recycling pathways through partially understood mechanisms (Joazeiro, 2017). ZNF598 is a human Really Interesting New Gene (RING) domain protein that shares homology with the yeast protein Hel2, the deletion of which promotes read-through of polybasic sequences (Brandman et al., 2012; Juszkiewicz and Hegde, 2017). A recent report has provided evidence that the E3-ligases Makorin 1 (MKRN1) and Makorin 2 (MKRN2) may complement the activity of ZNF598 in the ribosomal quality control pathway by promoting the initial stalling of the leading ribosome as it encounters a polyA tract (Hildebrandt et al., 2019).

The function of E3-ligases can be opposed by ∼100 deubiquitylase (DUB) enzymes drawn from seven families (Clague et al., 2019). RING E3s show a tendency to auto-ubiquitylate, leading to their destabilization, which can be rescued by the activity of interacting DUBs. The best known such example is provided by the association between USP7 and MDM2, which has rendered USP7 a prominent drug target, as a means to regulate levels of p53 (Li et al., 2004). Recent work focused on this enzyme has established proof of principle that selective small-molecule inhibition among the ubiquitin-specific protease (USP) family can be achieved (Gavory et al., 2018; Kategaya et al., 2017; Lamberto et al., 2017; Turnbull et al., 2017). USP9X is one of the most abundant members of the USP family and has been linked with many processes, including centrosome function, chromosome alignment during mitosis, EGF receptor degradation, chemo-sensitization, and circadian rhythms (Harris et al., 2012; Li et al., 2017; Savio et al., 2016; Wang et al., 2017; Zhang et al., 2018). Loss-of-function mutations in females lead to congenital malformations and intellectual disability (Reijnders et al., 2016). USP9X localization is predominantly cytosolic, but it can also influence events within the nucleus such as the DNA damage response (Murtaza et al., 2015; Nathan et al., 2008; O’Dea and Santocanale, 2020; Urbé et al., 2012).

In this study, we identify the E3 ligases ZNF598 and MKRN1/2 as USP9X binding partners and show that USP9X governs the stability of ZNF598 and Makorins. The loss or inhibition of USP9X leads to a substantive reduction in steady-state levels of ZNF598 and MKRN1/2 and disables an effective response to the presence of stalled ribosomes.

## Results and discussion

**Figure 1.**
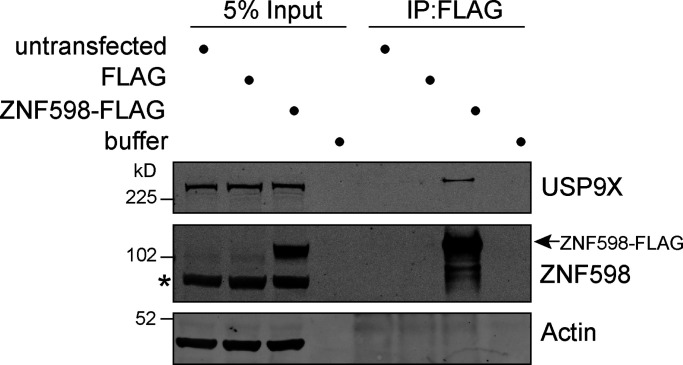
**USP9X coimmunoprecipitates with FLAG-tagged ZNF598.** HEK293T cells were transfected with ZNF598-FLAG or FLAG alone (pCMV-Tag2B), and cell lysates were subjected to immunoprecipitation (IP) with FLAG-antibody–coupled agarose beads. IPs were probed alongside 5% of the input as indicated, representative of two independent experiments. Arrow indicates ZNF598-FLAG; *, a nonspecific band.

In a large-scale proteomic study of the ribosome interactome, USP9X was one of only two DUB family members to be identified along with OTUD4 (Simsek et al., 2017). Furthermore, USP9X is also apparent within the set of ZNF598 interacting proteins, previously identified in a label-free proteomic study (Garzia et al., 2017). We sought to confirm this interaction by immunoprecipitating FLAG-tagged ZNF598 transiently expressed in HEK293T cells (Fig. 1). USP9X is clearly present in the immunoprecipitate containing ZNF598-FLAG and is absent from control lanes.

**Figure 2.**
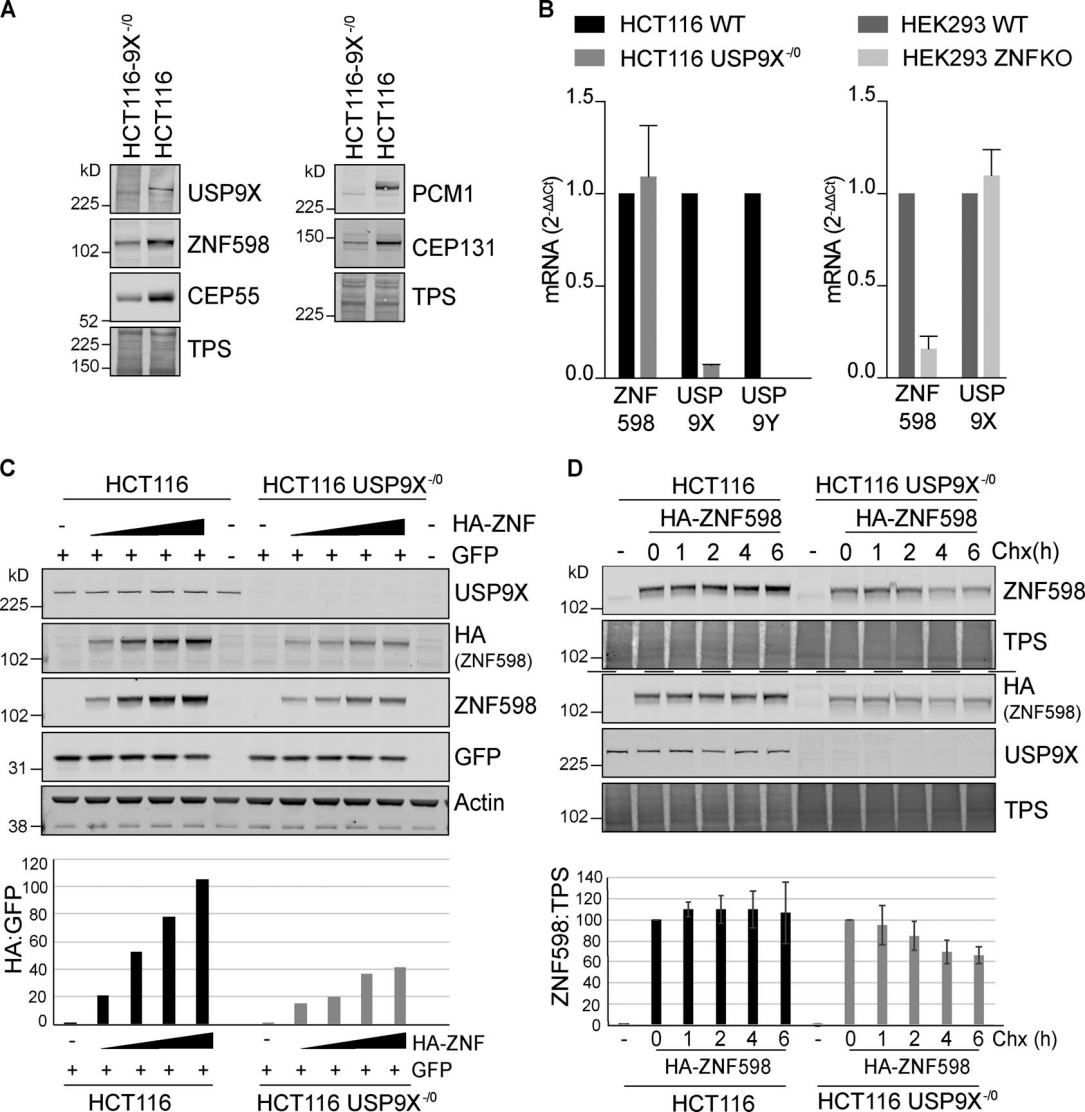
**ZNF598 is destabilized in USP9X KO cells. (A)** HCT116 or HCT116 USP9X^−^^/0^ lysates were analyzed by immunoblot with the indicated antibodies (representative of three independent experiments). **(B)** Quantitative RT-PCR reactions for ZNF598, USP9X, and USP9Y (normalized to Actin) were performed with cDNA derived from the indicated cell lines. The mean of three independent biological replicates is shown, and error bars indicate the standard deviation. **(C)** HCT116 or HCT116 USP9X^−^^/0^ cells were transfected with 0, 0.2, 0.4, 0.8, or 1.6 µg HA-ZNF598 and 0.2 µg GFP as a transfection control, and lysates analyzed by immunoblot with the indicated antibodies. Graph shows HA-ZNF598 relative to cotransfected GFP. Panel is representative of three experiments. **(D)** HCT116 or HCT116 USP9X^−^^/0^ cells were transfected with 0.2 µg HA-ZNF598 and treated for the indicated times with 100 µg/ml cycloheximide (Chx). Lysates (8 µg for HCT116 and 20 µg HCT116-USP9X^−^^/0^) were probed with the indicated antibodies. Graph represents the results from four independent experiments. Error bars represent the standard deviation. TPS, total protein stain.

**Figure S1.**
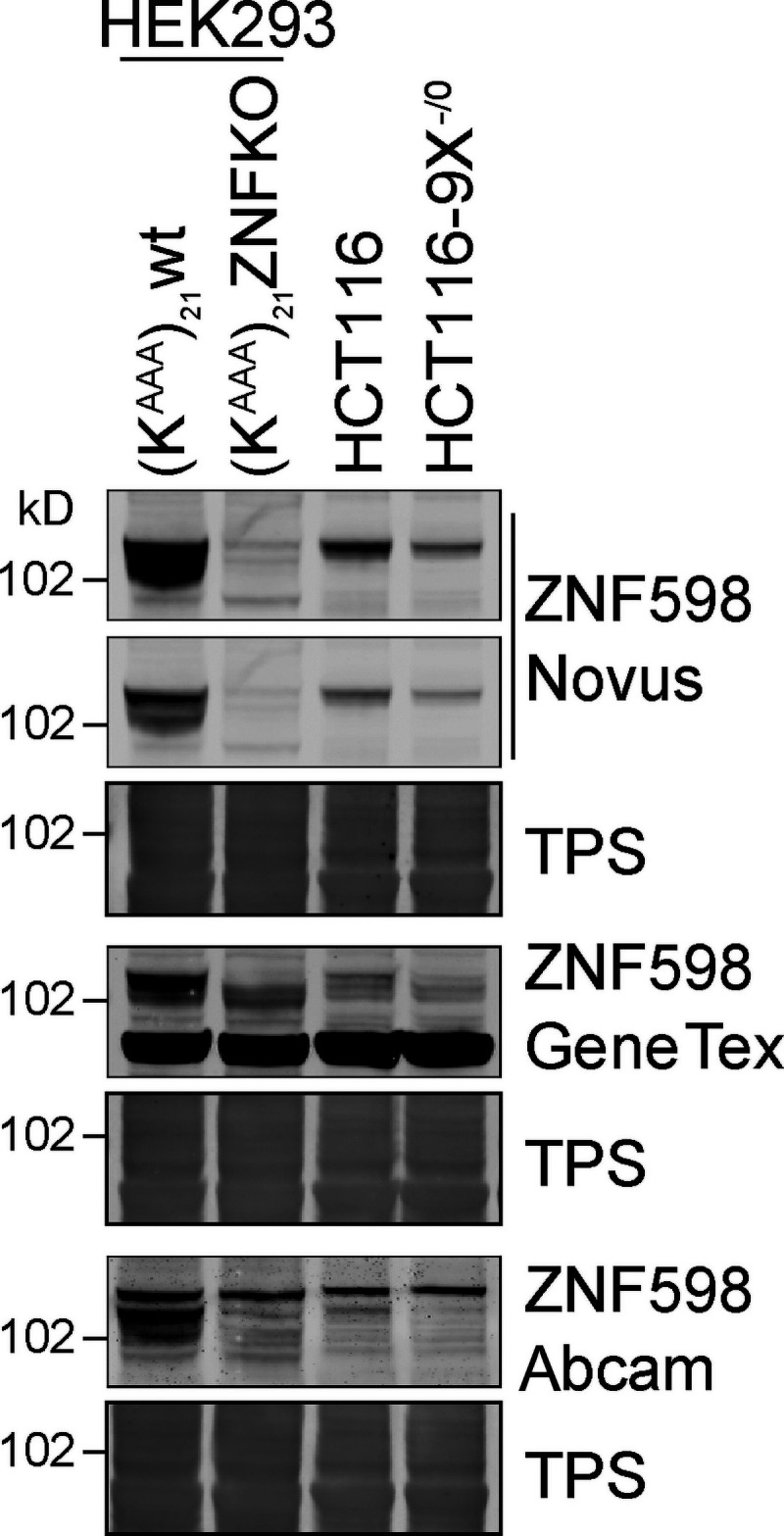
**ZNF598 antibody validation.** HEK293 Flp-In T-Rex GFP-P2A-(K^AAA^)_21_-P2A-RFP WT or ZNF598 KO, and HCT116 WT or HCT116 USP9X^−^^/0^ cell lysates were analyzed by immunoblotting with the indicated antibodies. TPS, total protein stain.

We next compared the engineered USP9X^−^^/0^ HCT116 colon cancer cells that have been described previously (Harris et al., 2012) with wild-type WT cells of the same origin. As expected, the USP9X^−^^/0^ cells show reduced levels of previously identified peri-centrosomal substrates CEP55, CEP131, and PCM1 (Li et al., 2017; Wang et al., 2017; Fig. 2 A). ZNF598 levels are also greatly diminished in these cells (Fig. 2 A and Fig. S1). Two lines of argument suggest that this is not an effect on transcription: (1) endogenous ZNF598 mRNA levels are similar between the two cell lines (Fig. 2 B), and (2) levels of exogenous HA-ZNF598 expression that is driven by a non-native promoter are also diminished in transfected cells (Fig. 2 C). We treated these cells with cycloheximide and monitored the decay of the expressed HA-ZNF598. In WT cells, levels of HA-ZNF598 remained stable over the 6 h of incubation, while in the USP9X^−^^/0^ cells, the levels significantly decline to ∼60% in the same period (Fig. 2 D). The most parsimonious explanation of these combined results is that USP9X interacts with ZNF598 and regulates its steady-state levels through the control of its stability.

**Figure 3.**
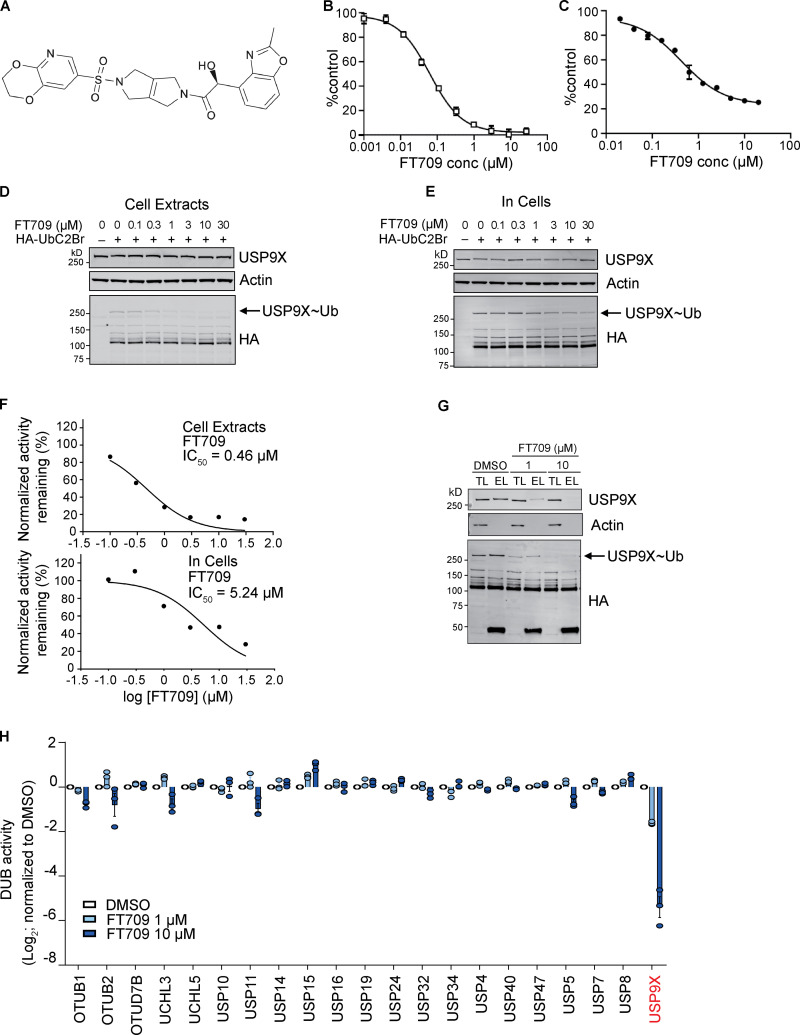
**Characterization of a highly selective USP9X inhibitor. (A)** Chemical structure of FT709. **(B)** In vitro potency of FT709 against USP9X. Activity is monitored by a fluorescence increase following cleavage of a Ub-rhodamine substrate. **(C)** BxPC3 cell-based potency of FT709 for reduction of CEP55 measured using a MSD ELISA assay. Graphs in B and C show the average of two experiments with error bars indicating the range. **(D–F)** Cell lysates (D) or intact MCF7 cells (E) were incubated with FT709 (30 min at 25°C for cell extracts, 3 h at 37°C for cells) at the indicated concentrations. Cells were lysed, and extracts were incubated with 0.1 µg HA-UbC2Br probe for 5 min at 37°C, followed by SDS-PAGE analysis. Samples were immunoblotted with USP9X and HA antibodies as indicated. Arrow indicates HA-probe labeled band corresponding to the USP9X~Ub probe adduct. Modification of USP9X with a ubiquitin probe (USP9X~Ub) was lost with increasing concentrations of inhibitor. **(F)** Quantitation of Western blots shown in D and E. **(G and H)** HA-based immunoprecipitation of HA-UbC2Br probe–labeled DUBs from cell lysates incubated first with DMSO, 1 or 10 µM FT709, for 1 h at 37°C. Immunoprecipitated proteins were eluted and either analyzed side by side with total lysate samples by immunoblotting (TL, total lysate; EL, eluate) or subjected to mass spectrometry–based quantification in three technical replicates. Differences in DUB-probe binding were quantified for 21 identified DUBs and normalized relative to DMSO control (error bars represent standard deviation of three technical replicates). See Fig. S2 for uncropped immunoblots. conc, concentration.

To demonstrate that stabilization of ZNF598 requires the catalytic activity of USP9X, we took advantage of a highly selective small-molecule inhibitor FT709 (Fig. 3). We identified USP9X inhibitors using a ubiquitin-TAMRA fluorescence polarization high-throughput screening assay, screening the inhibitory potential of a diverse collection of ∼140,000 compounds available at FORMA Therapeutics. Primary hits were further validated for direct USP9X binding by biophysical techniques such as surface plasmon resonance. Optimization of hits with respect to activity and physicochemical properties resulted in a series of compounds that includes FT709.

**Figure S2.**
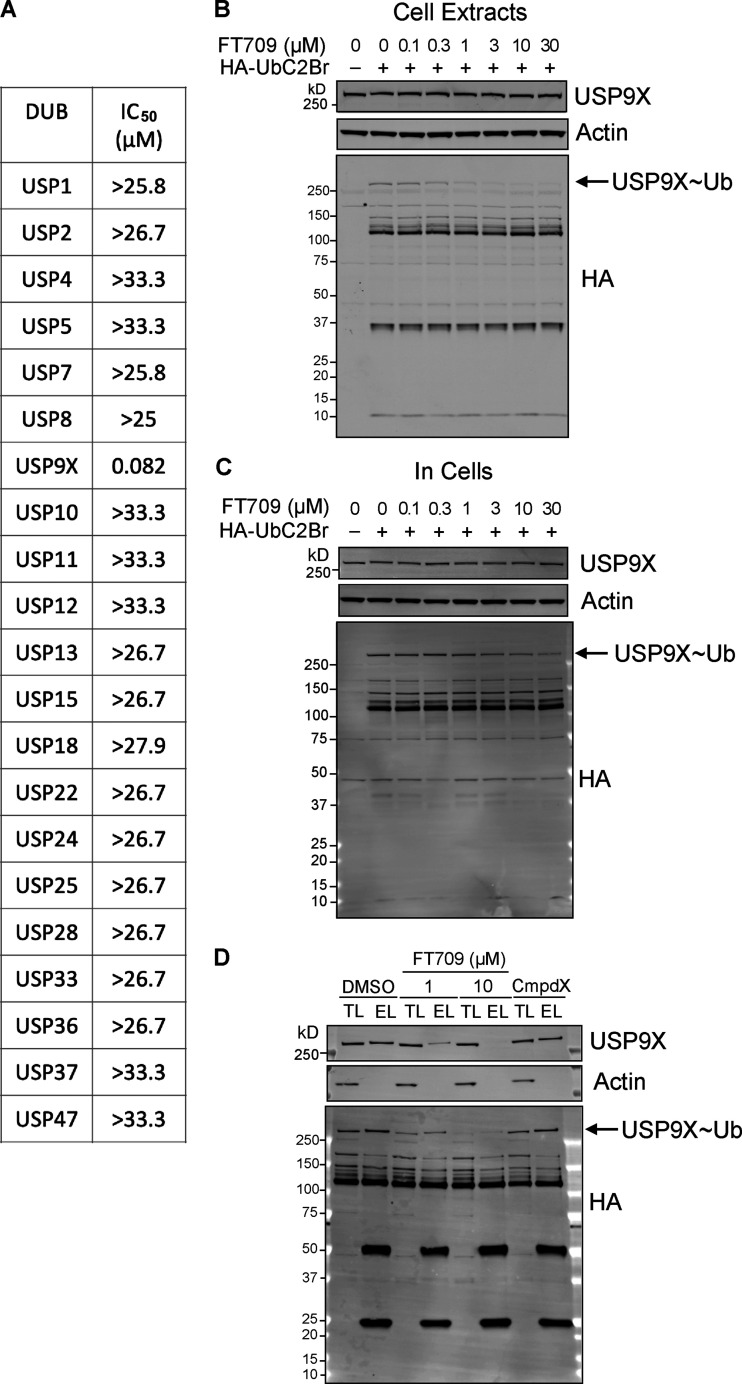
**Characterisation of FT709. (A)**IC_50_ values for FT709 inhibition across a panel of DUBs using an Ub-rhodamine as substrate. **(B–D)** Full Western blots of Fig. 3, D, E, and G. EL, eluate; TL, total lysate.

FT709 is potent in a biochemical assay with an half-maximal inhibitory concentration (IC_50_) of 82 nM (Fig. 3 B). Modulation of CEP55 expression in BxPC3 pancreatic cancer cells showed an IC_50_ of 131 nM (Fig. 3 C). The selectivity of FT709 was tested against >20 DUBs in a biochemical assay (Fig. S2 A) and was otherwise inactive across the panel (IC_50_ >25 µM). FT709 shows vastly improved specificity over the compound WP1130, which has previously been used as a USP9X inhibitor tool compound (Peterson et al., 2015; Ritorto et al., 2014). FT709 competes with an active site probe (HA-UbC2Br) with an IC_50_ of ∼0.5 µM and ∼5 µM when applied to MCF7 breast cancer cell extracts and to intact MCF7 cells, respectively (Fig. 3, D–F; and Fig. S2). Immunoprecipitation from cell lysates labeled with the active site probe HA-UbC2Br revealed that USP9X is uniquely sensitive to this compound, within a set of 21 DUBs quantified by mass spectrometry (Fig. 3, G and H; and Fig. S2).

**Figure 4.**
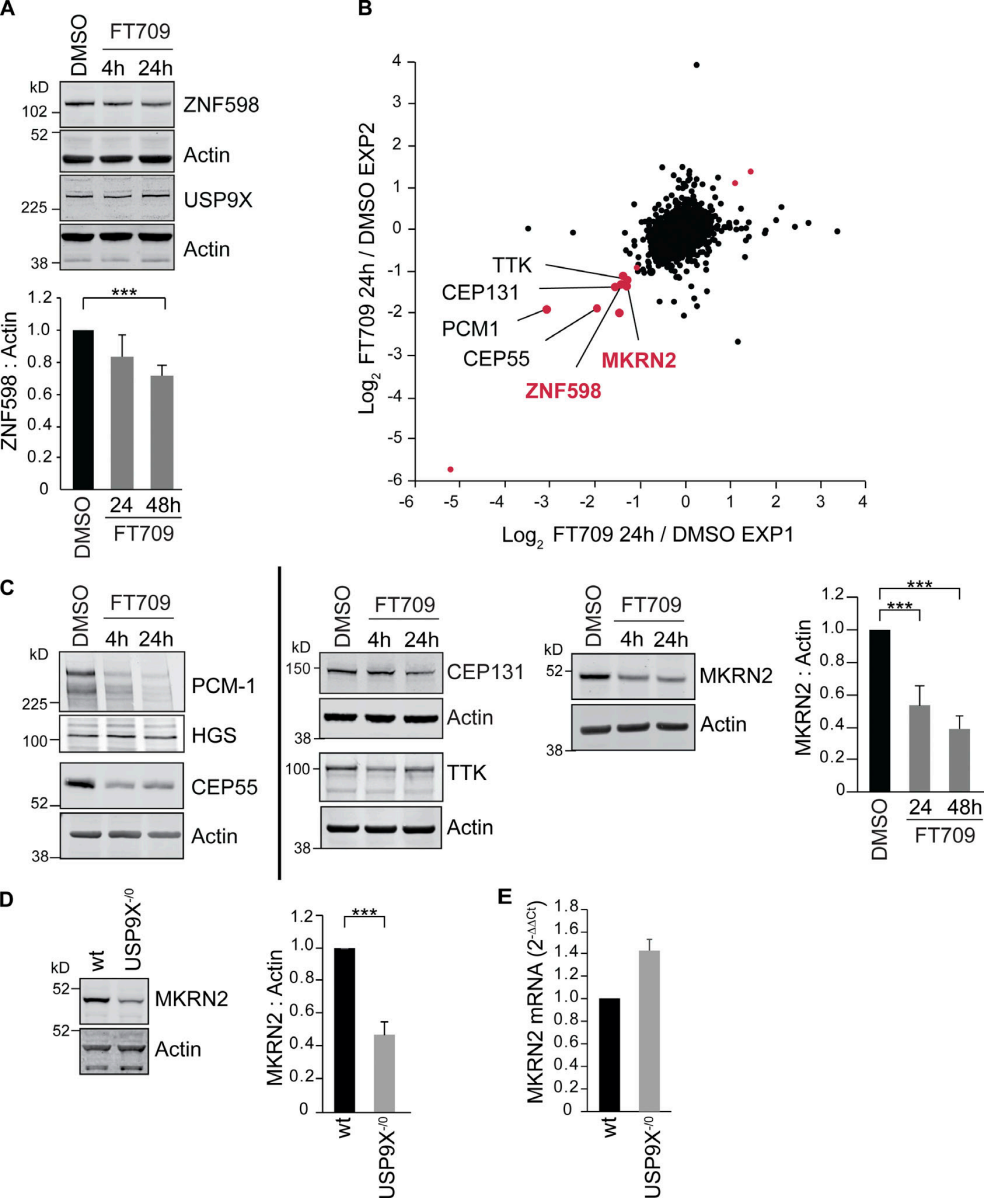
**Inhibition of USP9X catalytic activity depletes ZNF598 and MKRN2 protein levels. (A)** HCT116 cells were treated for 4 or 24 h with a selective USP9X inhibitor (FT709, 10 µM). Cells were lysed in RIPA buffer and samples analyzed by SDS-PAGE and immunoblotted for ZNF598 and USP9X. Graph represents quantification of four independent experiments. **(B)** Correlation of two distinct experimental SILAC-based proteomic datasets showing the de-enrichment of ZNF598 and MKRN2 alongside known USP9X substrates (in black type) in HCT116 cells treated for 24 h with USP9X inhibitor (FT709, 10 µM). Outliers for which the ratio is either lower than Log_2_(−1.0) or larger than Log_2_(+1.0) in both datasets are shown in red. **(C)** Western blot validation of USP9X inhibitor–sensitive proteins identified in B. HCT116 cells were treated with FT709 at 5 µM (CEP131), or 10 µM (all other samples) and analyzed as in A. Graph is representative of three independent experiments. **(D)** De-enrichment of MKRN2 in USP9X^−/0^ cells. HCT116 or HCT116 USP9X^−^^/0^ lysates were analyzed by immunoblot with the indicated antibodies. Quantification is based on four experiments. **(E)** Quantitative RT-PCR reactions for MKRN2 (normalized to actin) were performed with cDNA derived from the indicated HCT116 cell lines. *n* = 3 independent experiments. Error bars in all panels represent standard deviation; two-tailed Student’s *t* test; ***, P < 0.001.

**Figure S3.**
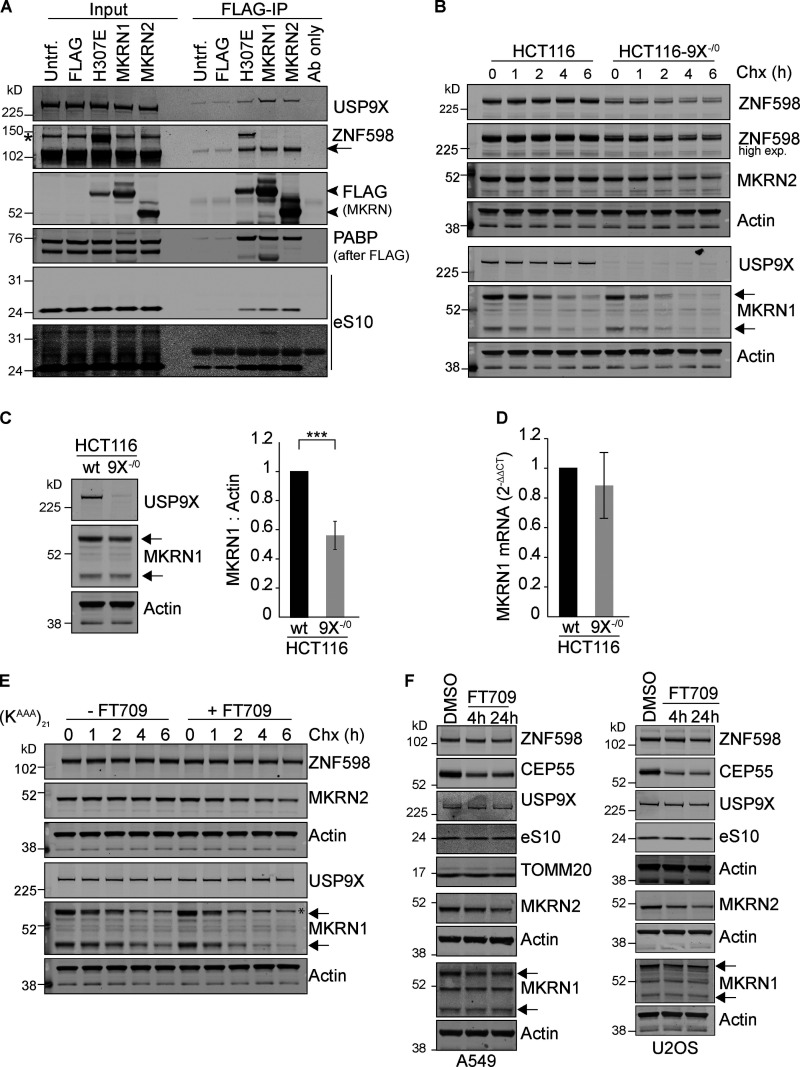
**Makorins interact with and are stabilised by USP9X. (A)** HEK293T cells were transfected with FLAG-MKRN1, FLAG-MKRN1-H307E, MKRN2-FLAG, or FLAG alone (pCMV-Tag2B) and cell lysates were subjected to immunoprecipitation (IP) with FLAG-antibody coupled agarose beads. IPs were probed alongside 2% of the input as indicated. Flag-MKRN1 H307E bears an inactivating mutation. PABP, polyA binding protein; eS10, 40S ribosomal subunit. Results are representative of three independent experiments. Arrow indicates ZNF598; arrowheads indicate FLAG-Makorins (MKRN). **(B)** HCT116 or HCT116 USP9X^−^^/0^ cells were treated for the indicated times with 100 µg/ml cycloheximide (Chx). **(C)** Steady-state levels of MKRN1 in HCT116 compared with HCT116 USP9X^−^^/0^ cells. Error bars indicate the standard deviation of four independent experiments, where the higher molecular weight isoform of MKRN1 has been quantified. **(D)** Quantitative RT-PCR reactions for MKRN1 (normalized to actin) were performed with cDNA derived from the indicated cell lines. The mean of three independent biological replicates is shown, and error bars indicate the standard deviation. **(E)** (K^AAA^)_21_ WT cells were treated for the indicated times with 100 µg/ml cycloheximide with or without FT709. **(F)** FT709 (10 µM) responsive markers in other cell types: A549 lung adenocarcinoma cells and U2OS osteosarcoma cells. TOMM20 here serves an alternative loading control for the upper set of panels. **(B, E, and F)** Arrows indicate two isoforms of MKRN1. *, a nonspecific band. Two-tailed Student’s *t* test; ***, P < 0.001. Untrf., untransfected.

Acute inhibition with FT709 recapitulates gene deletion of USP9X in HCT116 cells, leading to reduction of ZNF598 (Fig. 4 A). We conducted a wider survey of protein expression following USP9X inhibition through quantitative mass spectrometry (Fig. 4 B and Table S1). Among a small number of proteins that decrease by more than twofold following inhibitor treatment, known USP9X substrates are prominent. These include the (peri)-centrosomal proteins PCM1, CEP55, and CEP131 (Li et al., 2017; Wang et al., 2017) and the mitotic kinase TTK, also known as monopolar spindle 1 kinase (Fig. 4, B and C; Chen et al., 2018). ZNF598 is also found within this cohort, in alignment with our Western blot analysis (Fig. 4, A and B). Intriguingly, a second RING E3-ligase, MKRN2, which has been linked to ribosome stalling, is similarly identified as a clear outlier (Fig. 4, B and C; Hildebrandt et al., 2019). We tested the interaction between USP9X and Makorins by expression and immunoprecipitation of MKRN2-FLAG or FLAG-MKRN1 in HEK293 cells (Fig. S3 A). Both Makorins coimmunoprecipitate with USP9X, ZNF598, and the ribosomal subunit eS10. Accordingly, MKRN2 is reduced in USP9X^−^^/0^ cells with no corresponding reduction in mRNA levels (Fig. 4, D and E), reflecting decreased protein stability (Fig. S3 B). Note that ZNF598 and MKRN2 are the only RING E3-ligases contained within the proteomic dataset (>6,000 proteins) that show this magnitude of response to USP9X inhibition (Table S1). However, we could detect MKRN1 by Western blotting, and this is similarly destabilized by loss of USP9X in HCT116 cells (Fig. S3, B–D).

**Figure 5.**
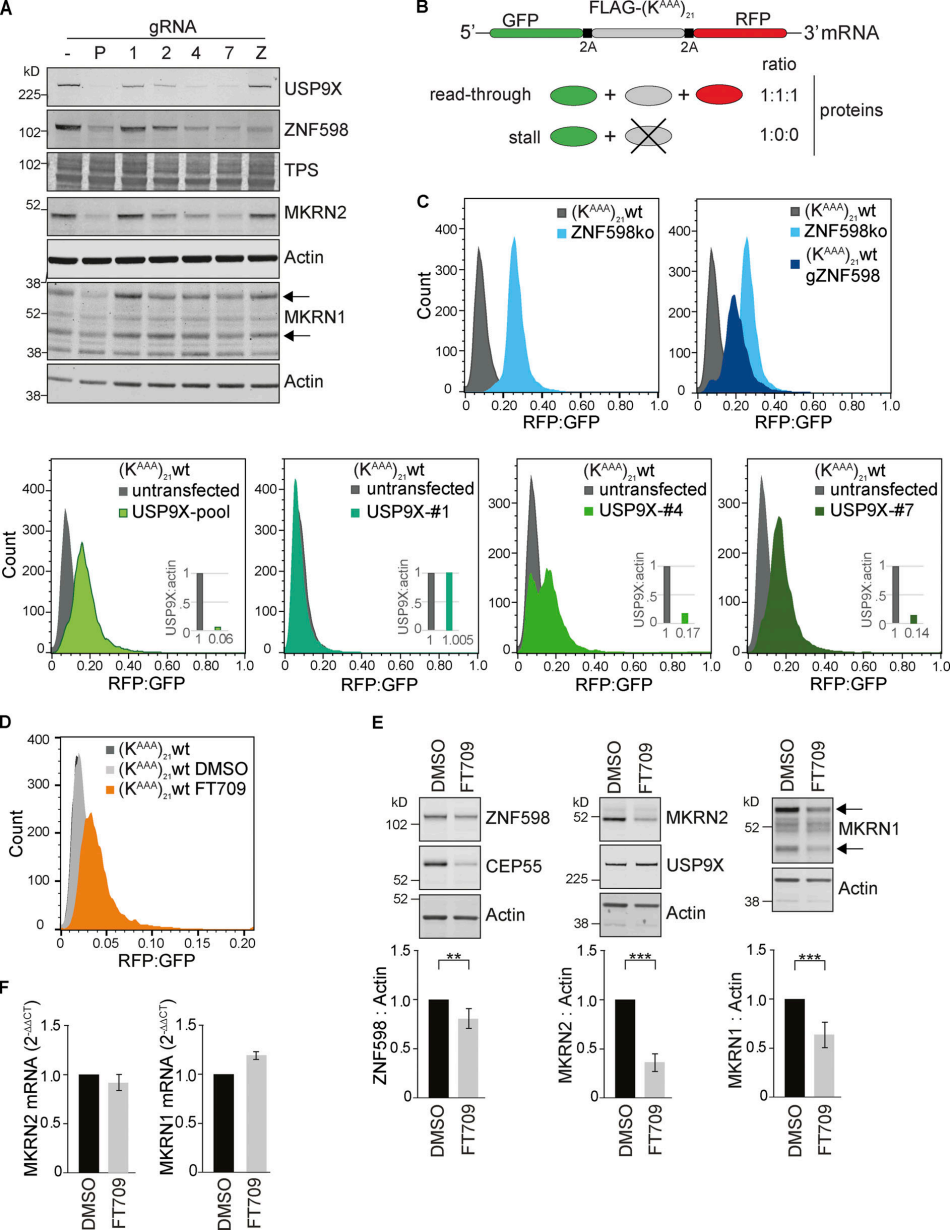
**USP9X ablation or inhibition impairs the ribosomal stalling response. (A)** HEK293 Flp-In T-Rex GFP-P2A-(K^AAA^)_21_-P2A-RFP WT cells were transfected with a plasmid containing Cas9 and gRNAs targeting USP9X or ZNF598. P, pool of USP9X guides; 1/2/4/7, individual USP9X guides; Z, ZNF598 guide. Lysates were analyzed 168 h after transfection and selection in puromycin by immunoblotting with the indicated antibodies. Panel is representative of two independent experiments. **(B)** Schematic of the fluorescent ribosomal stalling reporter expressed in this cell line. If stalling is not efficiently resolved, read-through occurs, and the FLAG-SR and RFP are expressed. **(C)** FACS analysis of the RFP:GFP ratio in (K^AAA^)_21_ WT or ZNF598 KO cells following transfection with px459-pSpCas9(BB)-2A-Puro_v2–containing gRNA as indicated. Cells were gated for live singlets, then for GFP-positive cells. Graphs depict data from >7,000 cells. Insets indicate the USP9X protein levels normalized to WT untransfected cells. **(D)** FACS analysis of the RFP:GFP ratio in WT cells following inhibition of USP9X with 10 µM FT709 for 72 h. Graph depicts data from >8,000 cells and is representative of three independent experiments. **(E)** (K^AAA^)_21_ WT cells were treated with indicated concentrations of FT709 for 48 h and analyzed by immunoblotting with selected antibodies (representative of three independent experiments). **(F)** quantitation of Makorin mRNA levels for cells treated as in E. Error bars in E and F indicate the standard deviation (*n* = 3 independent experiments); two-tailed Student’s *t* test; **, P < 0.01; ***, P < 0.001. Arrows in A and E indicate two isoforms of MKRN1, and the upper one is quantified. TPS, total protein stain.

We next used HEK293 cells to ask if the effects of USP9X ablation upon ZNF598 and MKRN2 could be extended to another cell type. We used a set of four individual gRNAs designed to target USP9X. They were inserted in an expression plasmid that also codes for Cas9 and a puromycin resistance cassette (px459-pSpCas9[BB]-2A-Puro-v2). Plasmids were transfected either individually or as a pool, and cell populations were harvested after 168 h of selection with puromycin. USP9X protein expression was effectively ablated in cells transfected with the pool of gRNAs, leading to a correspondingly stark reduction in ZNF598 and MKRN1/2 levels. ZNF598 loss, achieved through the same transient CRISPR/Cas9-based approach, showed no reciprocal effect on USP9X levels (Fig. 5 A), consistent with mRNA data presented in Fig. 2 B.

The HEK293 cells used in this study (HEK293-Flp-In TREX GFP-P2A-[K^AAA^]_21_ -P2A-RFP) have been engineered to express a reporter system for terminal ribosomal stalling (Juszkiewicz and Hegde, 2017). The reporter cassette contains GFP (N-terminal) and RFP (C-terminal) separated by a FLAG-tagged stalling reporter (SR) incorporating a polyA stretch of twenty-one codons (K^AAA^)_21_ (Fig. 5 B). This is flanked by viral P2A sequences, at which ribosomes skip formation of a peptide bond, without interrupting translation elongation. Consequently, unimpaired translation generates 3 proteins (GFP, FLAG-SR, RFP) in equal amounts. Stalling at the FLAG-SR aborts translation before RFP synthesis, leading to a substoichiometric RFP:GFP ratio. Failure to effectively respond to stalled ribosomes allows eventual read-through and a consequent rise in the RFP:GFP ratio that can be assessed by fluorescence-activated cell sorting (Fig. 5 B, schematic diagram; Juszkiewicz and Hegde, 2017). As reported previously, an isogenic reporter cell line, in which the ZNF598 gene has been deleted, shows an enhanced RFP:GFP ratio when compared with parental cells consistent with read-through, due to failure of the ribosomal stalling response (Juszkiewicz and Hegde, 2017). Using the pooled USP9X gRNA cells that show highly reduced levels of both USP9X and ZNF598, we could recapitulate this phenomenon (Fig. 5 C). We also analyzed three of the cell populations treated with individual gRNAs as represented in Fig. 5 A that show varying effectiveness for USP9X depletion. Guide 1 serves as a control because it was ineffective in editing the USP9X gene and accordingly shows no change in the RFP:GFP ratio. Guide 4 generates two distinct populations with ∼50% showing an enhanced RFP:GFP ratio, while Guide 7 shows a uniform enhancement of this ratio reminiscent of the effect of ZNF598 deletion and comparable to the pooled gRNA transfected sample (Fig. 5 C). Importantly, we were able to recapitulate polyA read-through with FT709, implicating USP9X enzymatic activity, and providing a pharmaceutical approach to counteract ribosomal stalling (Fig. 5 D). Chemical inhibition leads to a smaller reduction in ZNF598 levels but was again accompanied by parallel reductions in MKRN1 and 2, consequent to decreased protein stability (Fig. 5, E and F; and Fig. S3 E). We propose that USP9X inhibition may promote read-through by a combined effect on each of these RING E3 ligases linked to the ribosomal quality control pathway. Other cell types show a similar pattern of reduction in centrosomal and ribosomal stalling-associated proteins following application of FT709 (Fig. S3 F). In A549 and U2OS cells, FT709-dependent loss of ZNF598 is minor compared with MKRN2.

### Conclusions

USP9X is for the most part a nonessential DUB family member that is nevertheless expressed at relatively high levels (Behan et al., 2019; Clague et al., 2015). Multiple biological functions have been ascribed to USP9X that include roles in apoptosis, Wnt signaling, and mitotic checkpoint control (Nielsen et al., 2019; Schwickart et al., 2010; Skowyra et al., 2018). Our proteomics data most strongly support previously established links to centrosome biology (Han et al., 2019; Li et al., 2017; Wang et al., 2017). Here, we reveal a new biological role for USP9X in the resolution of stalled ribosomes, which is supported by unbiased proteomics. We propose that this is principally related to its governance of ZNF598 and Makorin ubiquitin E3 ligase levels (Hildebrandt et al., 2019). It is possible that USP9X could also play a more direct role in the deubiquitylation of ribosomal subunits themselves during resolution of stalling. However, this is difficult to unravel from effects upon their ubiquitin conjugation by the E3 ligases described here. Moreover, other DUBs (USP21, OTUD3) have recently been linked to this function (Garshott et al., 2020). The nonuniform dynamics of ribosomal processing, duration, and resolution of stalling may have important implications for protein folding, mRNA turnover, and the integrated stress response (Harding et al., 2019; Collart and Weiss, 2020). Recent studies have also shown that ribosomal collisions can result in +1 frame-shifting when the no-go RNA decay pathway is compromised (Simms et al., 2019). Our introduction of a highly specific USP9X tool compound inhibitor will enable further enquiry into pathways previously linked to USP9X, which should now include global profiling of protein translation.

## Materials and methods

### Chemical compound

FT709 used in this study was prepared by the procedures described in detail previously (Follows et al., 2020).

### Other materials

Antibodies were obtained from the following sources: rabbit anti-β-actin (Sigma-Aldrich; A2066); mouse anti-actin (Proteintech; 66009); rat anti-HA (Roche; 11867423001); rabbit anti-CEP131 (AZI1), rabbit anti-eS10 (RPS10, EPR8545), rabbit anti-MKRN2 (ab72055), rabbit anti-polyA binding protein (ab21060; all Abcam); rabbit anti-ZNF598 (Abcam, ab80458; Genetex, N1N3; Novus, NBP1-84659; all figures except Fig. 1 and Fig. 2 C); rabbit anti-CEP55 (D1L4H), rabbit anti-PCM-1 (G2000), rabbit anti-TTK (D15B7; all Cell Signaling Technology); mouse anti-FLAG (Sigma-Aldrich; F3165); goat anti-HGS (Everest Biotech; EB07211); rabbit anti-MKRN1 (A300-990A) and rabbit anti-USP9X (A301-350A; Bethyl Laboratories); mouse anti-TOMM20 (BD Transduction; B612278); and sheep anti-GFP (in-house). Infrared secondary antibodies were all from LI-COR Biosciences. Plasmids used were px459-pSpCas9(BB)-2A-Puro-v2 (Addgene; 62988), pcDNA3.1-ZNF598-TEV-3xFLAG (Addgene; 105690), pcDNA3 FLAG-MKRN1 (Addgene; 78751), pcDNA3 FLAG-MKRN1 H307B (Addgene; 78756). pcDNA3 MKRN2-FLAG was a gift from Chuanyin Li (Shanghai Instituet for Biological Sciences, Chinese Academy of Sciences, Shanghai, China). pCMV-HA-ZNF598 was generated in this study by subcloning the ZNF598 ORF from pcDNA3.1-ZNF598-TEV-3xFLAG into pCMV-HA (Clontech).

### Cell culture

HEK293T (gift from Maarit Suomalainen, University of Helsinki, Helsinki, Finland), U2OS, and A549 cells (ECACC) were cultured in DMEM with GlutaMAX + 10% FBS. HEK293 Flp-In 293 Trex K^(AAA)^^21^, WT, or ZNF598 knock-out (KO; Juszkiewicz et al., 2018; Juszkiewicz and Hegde, 2017) were cultured in DMEM with GlutaMAX + 10% tetracycline-free FBS, 15 µg/ml blasticidin, 100 µg/ml hygromycin. To induce reporter expression, 1 µg/ml doxycycline was added 24 h before harvesting. HCT116 and HCT116 USP9X^−^^/0^ were cultured in McCoys media + 10% FBS (Harris et al., 2012). MCF7 (ATCC) cells were cultured in DMEM medium supplemented with 10% FBS, 1% penicillin/streptomycin, and 1% glutamine at 37°C and 5% CO_2_. Cells were routinely checked for mycoplasma. For the cycloheximide assay, cells were treated for the indicated times with 100 µg/ml cycloheximide and harvested 24 h after transfection.

### Transfection

For transient transfection, 2 µg total DNA (per well of a six-well plate) was transfected using Genejuice (Novagen) according to the manufacturer’s instructions. Cells were harvested 24 or 168 h after transfection.

### Lysis and Western blot analysis

Cultured cells were lysed for 10 min at 4°C in RIPA buffer (10 mM Tris-HCl, pH 7.5, 150 mM NaCl, 1% Triton X-100, 0.1% SDS, and 1% sodium deoxycholate) supplemented with mammalian protease inhibitor cocktail (Sigma-Aldrich). Proteins were resolved using SDS-PAGE (Invitrogen NuPage gel 4–12%), transferred to nitrocellulose membrane, blocked in 5% fat-free milk or 5% bovine serum albumin in TBS supplemented with Tween-20, and probed with primary antibodies overnight. Visualization and quantification of Western blots were performed using IRdye 800CW and 680LT coupled secondary antibodies and an Odyssey infrared scanner (LI-COR Biosciences).

### Coimmunoprecipitation

Cells were lysed in TNTE buffer (10 mM Tris-Cl, pH 7.5, 150 mM NaCl, 0.3% Triton X-100, and 5 mM EDTA) supplemented with mammalian protease inhibitor cocktail (Sigma-Aldrich). 750 µg total protein was then incubated with 20 µg prewashed FLAG affinity gel (Sigma-Aldrich) for 2 h at 4°C, washed with TBS buffer (10 mM Tris-Cl, pH 7.5, and 100 mM NaCl), and eluted in sample buffer (62.5 mM Tris-Cl, pH 6.8, 3% SDS, 10% glycerol, and 3.2% β-mercaptoethanol). Immunoprecipitates were then analyzed by Western blot as above.

### RNA isolation and reverse transcription quantitative PCR

Total RNA was isolated from HCT116, HCT116 USP9X^−^^/0^, Flp-In 293 Trex K^(AAA)^_21_ WT, and Flp-In 293 Trex K^(AAA)^_21_ ZNF598 knockout (KO) using the Qiagen RNA extraction kit (74106). cDNA was generated using 1 µg RNA and the Thermo Scientific RevertAir H Minus reverse transcription (Thermo Fisher Scientific; 11541515) supplemented with RNasin (Promega; N251S), PCR nucleotide mix (Promega; U144B) and oligo (dT) 15 Primer (Promega; C1101). Quantitative PCRs were performed in triplicate using primers against ZNF598 (5′-GCT​CAT​CCA​GTC​CAT​CAG​GG-3′; 5′-GCA​GGA​CCA​GCA​GCT​CAT​TA-3′), MKRN1 (5′-CCA​ATG​GAT​GCT​GCC​CAG​AGA​T-3′; 5′-GGT​TGG​CTT​TCT​CAT​AGA​CCA​CC-3′), MKRN2 (5′-GGA​ACT​CGG​TGC​AGA​TAT​GAC-3′; 5′-GCA​GCT​GCC​TGG​ATT​ACT​C-3′), USP9X (5′-ACA​TGA​GTC​GCC​TCC​ACC​TG-3′; 5′-GCC​TGG​GTG​CAC​AGT​CTT​G-3′), USP9y (5′-ATG​AGC​CCT​CTC​CAT​CAG-3′; 5′-GAC​CTT​AGT​GCA​TAG​TCA​TAA​AG-3′), ACTB (5′-CAC​CTT​CTA​CAA​TGA​GCT​GCG​TGT​G-3′; 5′-ATA​GCA​CAG​CCT​GGA​TAG​CAA​CGT​AC-3′), and iTaq Mastermix (BioRad; 172–5171) in a BioRad CFX Connect real-time system. The mean cycle threshold (Ct) values were normalized to actin (ΔCt = Ct target − Ct actin), raised to the exponent of 2^−ΔCt^ and normalized to the respective WTWT control cell line to generate 2^−ΔΔCt^.

### FACS

sgRNAs targeting USP9X (1: 5′-CAC​CGA​TCA​ACA​GGC​CTC​GAT​GGG-3′; 2: 5′-CAC​CGA​TGC​TTC​ACT​TTT​AAC​ATC​A-3′; 4: 5′-CAC​CGA​TTC​TTG​CCA​TTG​AAG​GCA​C-3′; 7: 5′-CAC​CGA​TTC​ATG​TAA​CAA​GTA​GCA​C-3′) and ZNF598 (5′-CAC​CGT​AGA​GCA​GCG​GTA​GCA​CAC​C-3′) were cloned into px459-pSpCas9(BB)-2A-Puro-v2 vector at the BbsI site. These were transfected into Flp-In 293 Trex K^(AAA)21^ WT and ZNF598 KO cells (Juszkiewicz et al., 2018; Juszkiewicz and Hegde, 2017) using Genejuice (Novagen). 24 h after transfection, media were changed and 1 µg/ml puromycin included. Cells were then cultured for 7 d before harvesting for Western blot and FACS analysis. For FACS, cells were trypsinized, counted, resuspended in 10% tetracycline-free FBS in PBS, and analyzed on a FACS Aria III in conjunction with FlowJo software.

### DUB biochemical assay

The assay was performed in a final volume of 6 µl in assay buffer containing 20 mM Tris buffer, pH 8, 0.03% bovine γ-globulin, 0.01% Triton X-100, and 1 mM glutathione. Nanoliter quantities of 10-point, threefold serial dilutions in DMSO were predispensed into 1,536 assay plates for a final top concentration between 25 and 33.3 mM and subsequent half-log dilutions. 2× DUB (0.025 nM final concentration) was added and preincubated for 30 min at room temperature. 2× ubiquitin (Ub)-rhodamine (25 nM final concentration) was added to initiate the reaction. Fluorescence readings (excitation: 485 nm, emission: 535 nm) were acquired over 12 min (Envision reader). The slope of the data from each point was used to determine IC_50_.

For all assays, data were reported as percent inhibition compared with control wells. IC_50_ values were determined by curve fitting of the standard four-parameter logistic fitting algorithm included in the Activity Base software package IDBS XE Designer Model205. Data were fitted using the Levenburg–Marquardt algorithm.

### Meso Scale Discovery (MSD) assay for CEP55

BxPC3 cells were seeded in 96-well plates and exposed to FT709 (20 µM top concentration, 1:2 serial dilutions) for 6 h. Cell lysates were prepared in RIPA buffer and stored at −80°C until analysis. Samples were analyzed by an MSD ELISA assay (Pacific Biolabs) using a CEP55 antibody (Novux; 1:500 dilution in PBS) captured overnight at 4°C, 30 µl lysates per well, 30 µl of CEP55 antibody (CST; 81693) diluted 1:2,000 in 1% blocker A/PBS and 30 µl per well of a 1:4,000 diluted goat anti-rabbit sulfo-tag, 1% blocker A/PBS. Plates were read on a MSD Sector Imager 2400. Results were transformed as percentage DMSO controls and curves fitted using a nonlinear regression to determine the IC_50_. Results presented are based on duplicate values.

### DUB profiling assays using Ub-based active site-directed probes

Molecular probes based on the ubiquitin scaffold were generated and used essentially as described (Altun et al., 2011; McGouran et al., 2013). In brief, HA-tagged ubiquitin bromoethyl (HA-UbC2Br) was synthesized by expressing the fusion protein HA-Ub_75_-intein-chitin–binding domain in *Escherichia coli* BL21 strains (Borodovsky et al., 2002). Bacterial lysates were prepared and the fusion protein purified over a chitin-binding column (NEB). HA-Ub_75_-thioester was obtained by incubating the column material with mercaptosulfonate sodium salt overnight at 37°C. HA-Ub_75_-thioester was concentrated to ∼1 mg/ml using 3,000 molecular weight filters (Sartorius) and then desalted against PBS using a PD10 column (GE Healthcare). 500 µl of 1–2 mg/ml of HA-Ub_75_-thioester was incubated with 0.2 mmol of bromo-ethylamine at pH 8–9 for 20 min at room temperature, followed by a desalting step against phosphate buffer, pH 8.0, as described above. Ub probe material was concentrated to ∼1 mg/ml, using 3,000 molecular weight filters (Sartorius), and kept as aliquots at −80°C until use.

### DUB competition assays in cell extracts and in cells (in situ)

Crude MCF7 cell extracts were prepared as described previously using glass-bead lysis in 50 mM Tris, pH 7.4, 5 mM MgCl_2_, 0.5 mM EDTA, 250 mM sucrose, and 1 mM DTT (Borodovsky et al., 2002; McGouran et al., 2013). For experiments with crude cell extracts, 50 µg of MCF7 cell lysate was incubated with different concentrations of FT709 for 1 h at 37°C, followed by addition of 1 µg HA-UbC2Br and incubation for 5 min at 37°C. Incubation with Ub-probe was optimized to minimize replacement of noncovalent inhibitor FT709 by the covalent probe. Samples were then subsequently boiled in reducing SDS sample buffer, separated by SDS-PAGE, and analyzed by Western blotting using anti-HA (1:2,000), anti-USP9X (Cell Signaling Technology; D4Y7W 14898 at 1:1,000), or β-actin (1:2,000) antibodies. 5 × 10^6^ intact MCF7 cells were incubated with different concentrations of inhibitors in cultured medium for 4 h at 37°C, followed by glass bead lysis, labeling with HA-UbC2Br probe, and analysis by SDS-PAGE and Western blotting as described above.

### Mass spectrometry–based DUB inhibitor profiling assays

Ub-probe pulldown experiments in the presence of different concentrations of the inhibitor FT709 were performed essentially as described (Altun et al., 2011; McGouran et al., 2013) with some modifications. In brief, immunoprecipitated material from 500 µg to 1mg of MCF-7 cell crude extract was subjected to in-solution trypsin digestion and desalted using C18 SepPak cartidges (Waters) based on the manufacturer’s instructions. Digested samples were analyzed by nano-ultra performance liquid chromatography (UPLC)-mass spectrometry (MS)/MS using a Dionex Ultimate 3000 nano UPLC with EASY spray column (75 µm × 500 mm, 2 µm particle size; Thermo Fisher Scientific) with a 60-min gradient of 0.1% formic acid/5% DMSO to 0.1% formic acid/35% acetonitrile/5% DMSO at a flow rate of ∼250 nl/min (∼600 bar/40°C column temperature). MS data were acquired with an Orbitrap Q Exactive High Field (HF) instrument in which survey scans were acquired at a resolution of 60,000 at 400 m/z, and the 20 most abundant precursors were selected for collision-induced dissociation fragmentation. From raw MS files, peak list files were generated with MSConvert (Proteowizard V3.0.5211) using the 200 most abundant peaks/spectrum. The Mascot (V2.3, Matrix Science) search engine was used for protein identification at a false discovery rate of 1%, mass deviation of 10 ppm for MS1 and 0.06 D (Q Exactive HF) for MS2 spectra, Cys carbamidylation as fixed modification, Met oxidation, and Gln deamidation as variable modification. Searches were performed against the UniProtKB human sequence database (retrieved October 15, 2014). Label-free quantitation was performed using MaxQuant Software (version 1.5.3.8), and data were further analyzed using GraphPad Prism software (v7) and Microsoft Excel.

### Stable isotope labeling by amino acids in cell culture (SILAC)-based proteome analysis of FT709-treated HCT116 cells

HCT116 cells were grown in SILAC DMEM supplemented with 10% dialyzed FBS (Dundee Cell Products) at 37°C and 5% CO_2_. To generate light, medium, and heavy stable isotope-labeled cells, arginine- and lysine-free DMEM medium was supplemented with 200 mg/liter L-proline and either L-lysine (Lys0) together with L-arginine (Arg0; light), L-lysine-^2^H_4_ (Lys4) with L-arginine-U-^13^C_6_ (Arg6; medium), or L-lysine-U-^13^C_6_-^15^N_2_ (Lys8) with L-arginine-U-^13^C_6_-^15^N_4_ (Arg10; heavy) at final concentrations of 84 mg/liter for the arginine and 146 mg/liter for the lysine until fully metabolically labeled. Cells were treated with DMSO or 10 µM FT709 for 4 h or 24 h, before lysis in 50 mM Tris, pH 6.8, 2% SDS, and 10% glycerol. Relative protein concentrations of the lysates were determined using a bicinchoninic acid assay (Thermo Fisher Scientific), and light-, medium-, and heavy-labeled lysates were combined in a 1:1:1 ratio.

### Deep proteome workflow

Protein extracts (1.2–1.5 mg) containing SDS were reduced with 5 mM dithiothreitol, alkylated with 20 mM iodoacetamide, and then subjected to methanol/chloroform extraction. Protein pellets were resuspended in 6 M urea by vortexing and sonication, then diluted to a final concentration of 1 M before in-solution digestion with 0.2 µg/µl trypsin (sequencing grade, Promega) overnight at 37°C. Off-line high-pH reverse-phase prefractionation was performed on the digested material as previously described (Davis et al., 2017), with the exception that eluted peptides were concatenated down to 10 fractions. Peptide fractions were analyzed in technical replicates by nano-UPLC-MS/MS using a Dionex Ultimate 3000 nano UPLC with EASY spray column (75 µm × 500 mm, 2 µm particle size; Thermo Fisher Scientific) with a 60-min gradient of 2% acetonitrile/0.1% formic acid/5% DMSO to 35% acetonitrile/0.1% formic acid/5% DMSO at a flow rate of ∼250 nl/min. MS data were acquired with an Orbitrap Q Exactive HF instrument in which survey scans were acquired at a resolution of 60,000 at 200 m/z, and the 20 most abundant precursors were selected for higher-energy collisional dissociation fragmentation with a normalized collision energy of 28.

### Data analysis

All raw MS files from the biological replicates of the SILAC-proteome experiments were processed with the MaxQuant software suite, version 1.5.3.8, using the Uniprot database (uniprotHumanUP000005640.fasta, retrieved July 2015) and the default settings (Tyanova et al., 2016). The minimum required peptide length was set to six amino acids, and two missed cleavages were allowed. Cysteine carbamidomethylation was set as a fixed modification, whereas oxidation and N-terminal acetylation were considered as variable modifications. ProteinGroup text files were further processed using Excel (see Table S1), and the log_2_ of the normalized ratios was plotted using JMP software (version 13.0.0). The mass spectrometry proteomics data have been deposited to the ProteomeXchange Consortium via the PRIDE partner repository with the dataset identifier PXD018662.

### Online supplemental material

Fig. S1 shows additional Western blot validation data for ZNF598 antibodies, supplementing Fig. 2. Fig. S2 shows IC_50_ data for FT709 across a panel of DUBs and the uncropped version of HA immunoblots shown in Fig. 3, D, E, and G. Fig. S3 shows supplementary data supporting the interaction between USP9X, and MKRN1 and 2; and cycloheximide chase and quantitative RT-PCR data supporting a role for USP9X in the stabilization of MKRN1 and 2. Table S1 shows proteomic data for Fig. 4 B.

## Supplementary Material

Table S1 shows proteomic data for Fig. 4 B.
