# Correction: The deubiquitylase USP9X controls ribosomal stalling

**DOI:** 10.1083/jcb.20200421102102021c

**Published:** 2021-02-15

**Authors:** Anne Clancy, Claire Heride, Adán Pinto-Fernández, Hannah Elcocks, Andreas Kallinos, Katherine J. Kayser-Bricker, Weiping Wang, Victoria Smith, Simon Davis, Shawn Fessler, Crystal McKinnon, Marie Katz, Tim Hammonds, Neil P. Jones, Jonathan O'Connell, Bruce Follows, Steven Mischke, Justin A. Caravella, Stephanos Ioannidis, Christopher Dinsmore, Sunkyu Kim, Axel Behrens, David Komander, Benedikt M. Kessler, Sylvie Urbé, Michael J. Clague

Vol. 220 No. 3 | 10.1083/jcb.202004211 | January 28, 2021

After publication, the authors reported a mistake made during the assembly of the Western blots in Fig. 4 D, which occurred after peer review and prior to publication. The labels “wt” and “USP9X^−/0^” were mistakenly swapped. Reviewers and editors had assessed the correct version of the figure. The conclusions drawn from the experiment shown in Fig. 4 D are not affected.

**Figure:**
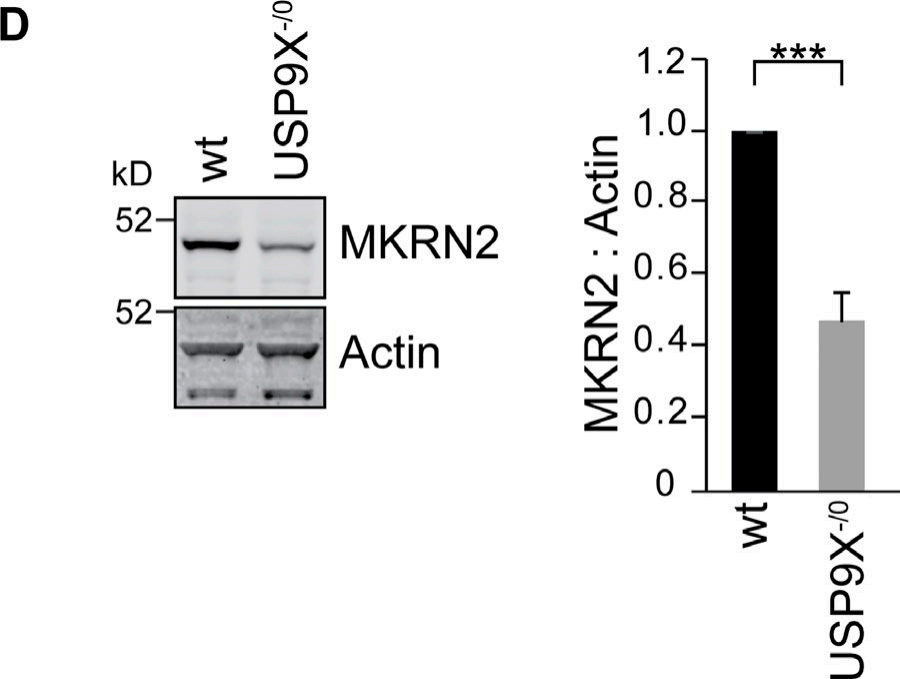


The labels in the Western blots of Fig. 4 D have been adjusted accordingly, and the corrected figure panel is shown here. This error appears only in print and in PDF versions downloaded on or before February 12, 2021.

